# Supplementary material for: Soluble pathogenic tau enters brain vascular endothelial cells and drives cellular senescence and brain microvascular dysfunction in a mouse model of tauopathy
Source: Nat Commun. 2023 Apr 25;14:2367. doi: 10.1038/s41467-023-37840-y (PMC10126555; doi:10.1038/s41467-023-37840-y)
Supplement: Supplementary file 1 — Supplementary Information [file 41467_2023_37840_MOESM1_ESM.pdf]

Antibody	Supplier	Catalog #	Lot	Clone	Species	Application/Dilution		
						IF/ICC Dilution	Traditional WB	CE Immunblots
β-Actin	Cell Signaling Technology	4970S	15	13E5	Rabbit		1:1000	1:200
CD31	BD Biosciences	553370	2146504	MEC 13.3	Mouse	1:500		
eNOS	R&D	AF950	BBu0417051		Goat	1:500		
phospho-eNOS (S1176/1177)	BD Biosciences	612392	9044662	19/eNOS/S1177	mouse			1:50
phospho-eNOS (T495)	BD Biosciences	612706	5329915	31/eNOS(pT495)	Mouse			1:50
eNOS	Millipore	07-520	2708538		Rabbit			1:75
Fibrinogen	Dako Agilent	A0080	15063		Rabbit	1:750		
GAPDH	Thermo Fisher Scientific	PA1-987	UG28627		Rabbit		1:1000	
Tau5	Millipore	MAB361	3275077	Tau-5	Mouse	1:500	1:500	1:50
phospho-Tau (T231)	Thermo Scientific,	MN1040	PF202799	AT180	Mouse			1:50
phospho-Tau AT8	Thermo Fisher Scientific	MN1020	VE2953712	AT8	Mouse	1:500		
T22 (Oligomeric tau)	Dr. Rakez Kayed Lab				Rabbit	1:500		
α-tubulin	Cell Signaling Technology	2144S	6		Rabbit			1:1000
Acetyl-α-Tubulin	Santa Cruz Biotechnology	sc-23950	F1516	6-11B-1	Mouse	1:500		1:2000
β-tubulin	Sigma Aldrich	T4026	107M4801V	TUB2.1	Mouse	1:1000		
α/β tubulin	Cell Signaling Technology	2148S	8		Rabbit	1:1000		
V5 antibody	Thermo Fisher Scientific	R960-25	2024280	P/N 46-0705	Mouse	1:500		
Goat anti-Rabbit IgG (H+L) Cross-Adsorbed Secondary Antibody, Alexa Fluor™ 594	Invitrogen	A11012	1933366		Goat	1:500		
Goat anti-Mouse IgG (H+L) Highly Cross-Adsorbed Secondary Antibody, Alexa Fluor™ 488	Invitrogen	A11029	2120125		Goat	1:500		
Goat anti-Mouse IgG (H+L) Highly Cross-Adsorbed Secondary Antibody, Alexa Fluor™ 647	Invitrogen	A21236	1229707		Goat	1:500		
Donkey anti-Goat IgG (H+L) Highly Cross-Adsorbed Secondary Antibody, Alexa Fluor™ Plus 488	Invitrogen	A32814	UI289709		Donkey	1:500		

Supplementary Table 1. Antibody Information. Supplier, catalog number, lot number, clone, and dilution information for antibodies used in immunofluorescence (IF), immunocytochemistry (ICC), tradition Western blot (WB), and capillary electrophoresis (CE) experiments.

Species	Gene Transcript	5' → 3'	
		Forward	Reverse
Human	Tau	CCTCTCCCGTCCTCGCCTCTG	GGGTCAGCCATCCTGGTTCA
Human	Cdkn2a	ACTTCAGGGGTGCCACATTC	CGACCCTGTCCCTCAAATCC
Human	Cdkn1a	AGTCAGTTCCTTGTGGAGCC	CATTAGCGCATCACAGTCGC
Human	Tp53	CCTGGATTGGCCAGACTGC	TTTTCAGGAAGTAGTTTCCATAGGT
Human	IL-6	TGAACTCCTTCTCCACAAGCG	GAGATGCCGTCGAGGATGTA
Human	IL-1β	CAGAAGTACCTGAGCTCGCC	AGATTCGTAGCTGGATGCCG
Human	TNFα	CACAGTGAAGTGCTGGCAAC	AGGAAGGCCTAAGGTCCACT
Human	PAI-1	CCTCAGGAAGCCCCTAGAGA	ATGCGGGCTGAGACTATGAC
Human	MCP-1	TTCCCCTAGCTTTCCCCAGA	TCCCAGGGGTAGAACTGTGG
Human	GAPDH	GGTGACTAACCCTGCGCTC	CAGAGTTAAAAGCAGCCCTGG
Mouse	Cdkn2a	CGAACTCGAGGAGAGCCATC	GGAGAAGGTAGTGGGGTCCT
Mouse	Cdkn1a	GTGGGTCTGACTCCAGCCCC	CCTTCTCGTGAGACGCTTAC
Mouse	Tp53	CACAGCGTGGTGGTACCTTA	TCTTCTGTACGGCGGTCTCT
Mouse	IL-6	CCGGAGAGGAGACTTCACAG	TCCACGATTTCCCAGAGAAC
Mouse	IL-1β	GAGCACAAGCCTGTCTTCCT	GGCCGAGGACTAAGGAGTCT
Mouse	TNFα	CGGGCAGGTCTACTTTGGAG	ACCCTGAGCCATAATCCCCT
Mouse	PAI-1	GACACCCTCAGCATGTTCATC	AGGGTTGCACTAAACATGTCAG
Mouse	MCP-1	GCTCAGCCAGATGCAGTTAA	TCTTGAGCTTGGTGACAAAAACT
Mouse	GAPDH	ACCAACTGCTTAGCCCCCC	TGCAGGGATGATGTTCTGGG

Supplementary Table 2. qRT-PCR Primer Sequences

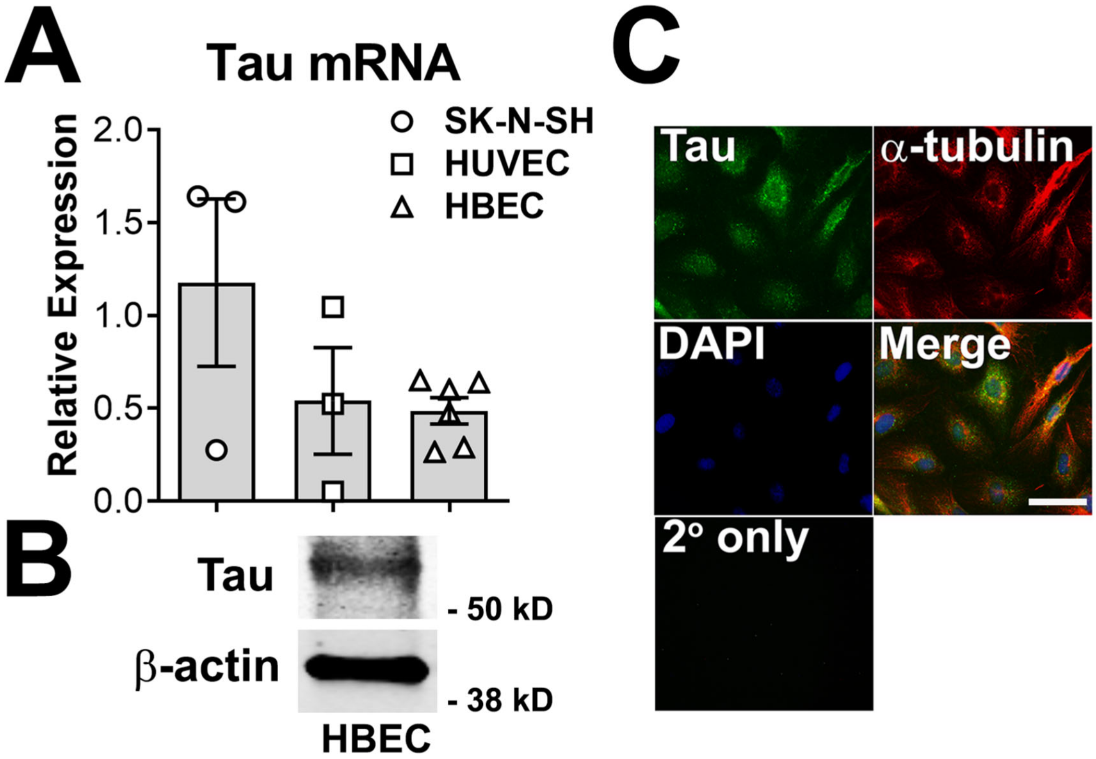

Supplementary Figure 1. Tau is expressed in primary human brain microvascular endothelial cells and localizes in close proximity to microtubules. **(A)** Quantitative real-time PCR measurements of tau mRNA abundance in human SK-N-SH neuroblastoma cells, human brain microvascular endothelial cells (HBEC), and primary human umbilical vein endothelial cells (HUVEC) show comparable expression of tau in cells of endothelial and neuronal origin ($F(2, 9) = 2.467$, ANOVA, $p=0.14$. SK-N-SH, n=3; HUVEC, n=3; HBEC, n=6 biologically independent samples examined in 1 independent experiment for SK-N-SH and HUVEC, 3 independent experiments for HBEC). Data are means ± SEM. **(B)** Western blot showing tau protein content in HBEC with β-actin as reference, n=1 biologically independent sample examined in 1 independent experiment. **(C)** Tau immunoreactivity in HBEC (Tau5, green) is found in close proximity to tubulin (α/β-tubulin, red). Scale bar is 50 μm.

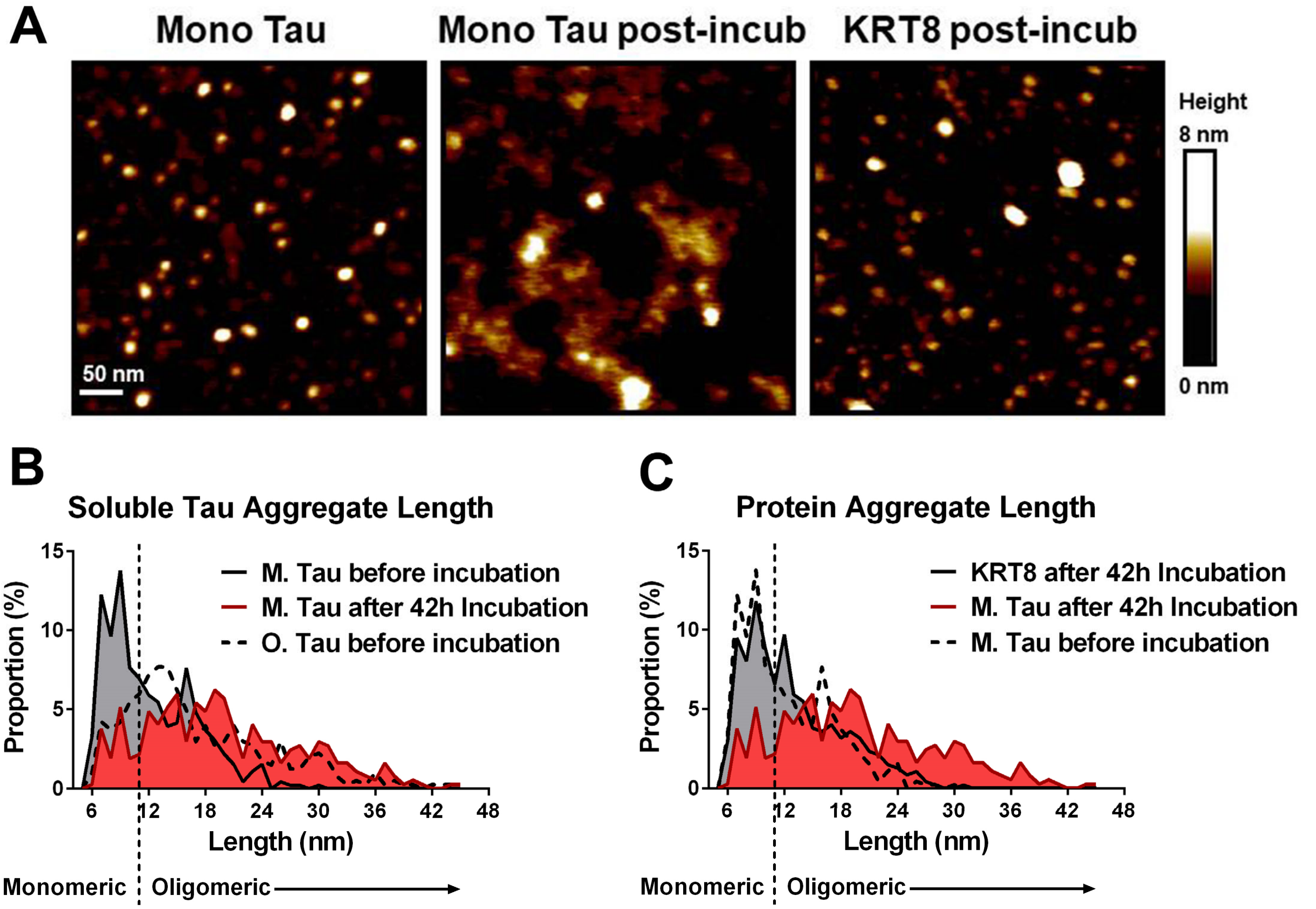

Supplementary Figure 2. Tau monomers spontaneously oligomerize during incubation with cultures of human brain microvascular endothelial cells (HBEC). A, Representative AFM images of field fragments with monomeric tau, monomeric tau post-incubation with HBEC, and human cytokeratin-8 (KRT8) post-incubation with HBEC. **B,** Length profiles (nm) of tau species measured by atomic force microscopy in media from HBEC cultures treated with tau monomers (M. Tau) before and after 42 h of incubation (M. Tau before incubation, n=458 particles measured; M. Tau after incubation, n=370 particles measured). Length profiles of tau species in media with tau oligomers (O. Tau) are provided as reference (O. Tau, n=403, Figure 1F). **C,** Length profiles (nm) of human cytokeratin-8 (KRT8, gray) and monomeric tau (M. Tau, red) measured by atomic force microscopy. Monomeric tau was measured in media before and after, and KRT8 was measured in media after 42 hours of incubation with HBEC (M. Tau before incubation, n=458 particles measured (also in Figure S1B); M. Tau after incubation, n=370 particles measured (also in Figure S1B); KRT8 after incubation, n=474). Length profiles of tau in media spiked with tau monomer preparations (M. Tau, dashed line) are

provided as reference. Particles measured were examined over 1 independent experiment. Data are representative images and frequency profiles.

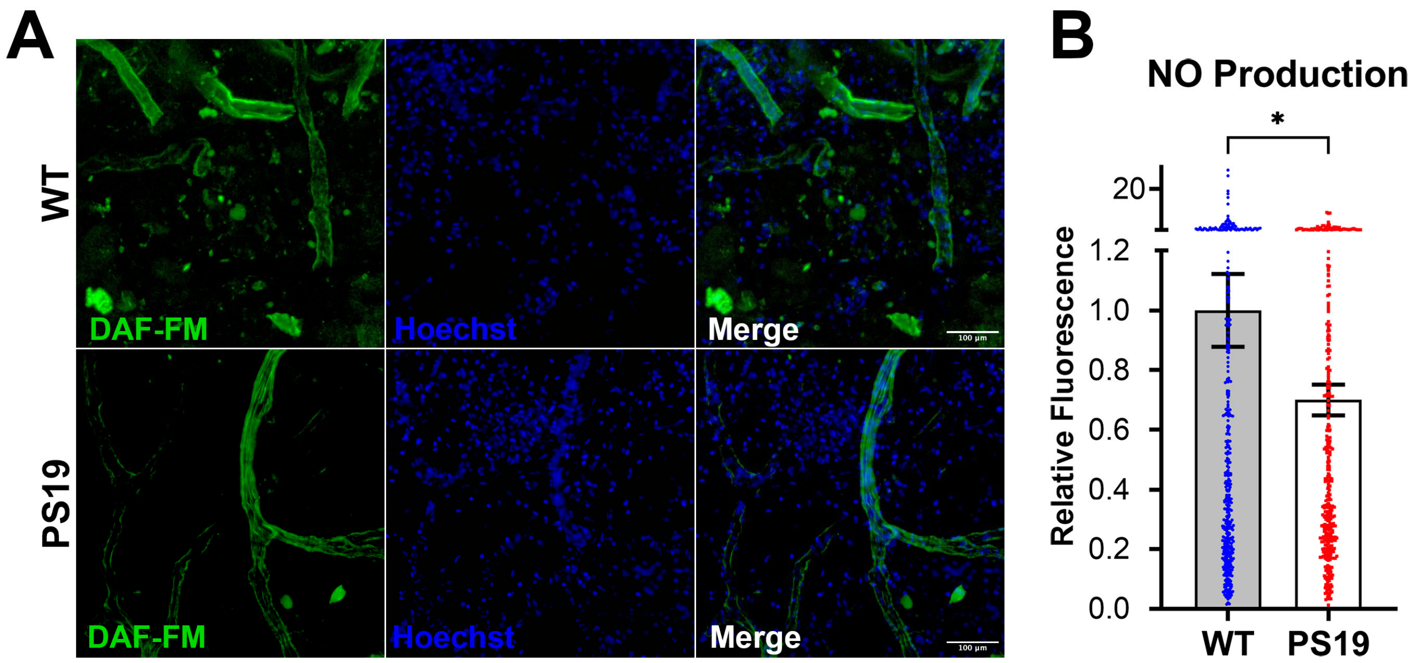

Supplementary Figure 3. Decreased nitric oxide production in microvessels isolated from P301S(PS19) mice. A, Brain microvessels isolated from three individual male and female 9-month-old WT and three individual 9-month-old P301S(PS19) mice incubated with DAF-FM, a nitric oxide indicator (green), and Hoechst 33342, a nuclear stain (blue). Scale bar is 100 μm. **B,** Quantification of DAF-FM fluorescence normalized to vessel area and diameter for all microvessels from each animal. Unpaired, two-sided Student's t test with Welch's correction ($t(580.9)=2.265$, *, $P=0.024$. WT, $n=436$; PS19, $n=372$ individual vessel segments obtained from 3 WT and 3 PS19 mice, examined in 2 independent experiments). Data are representative images and means ± SEM.

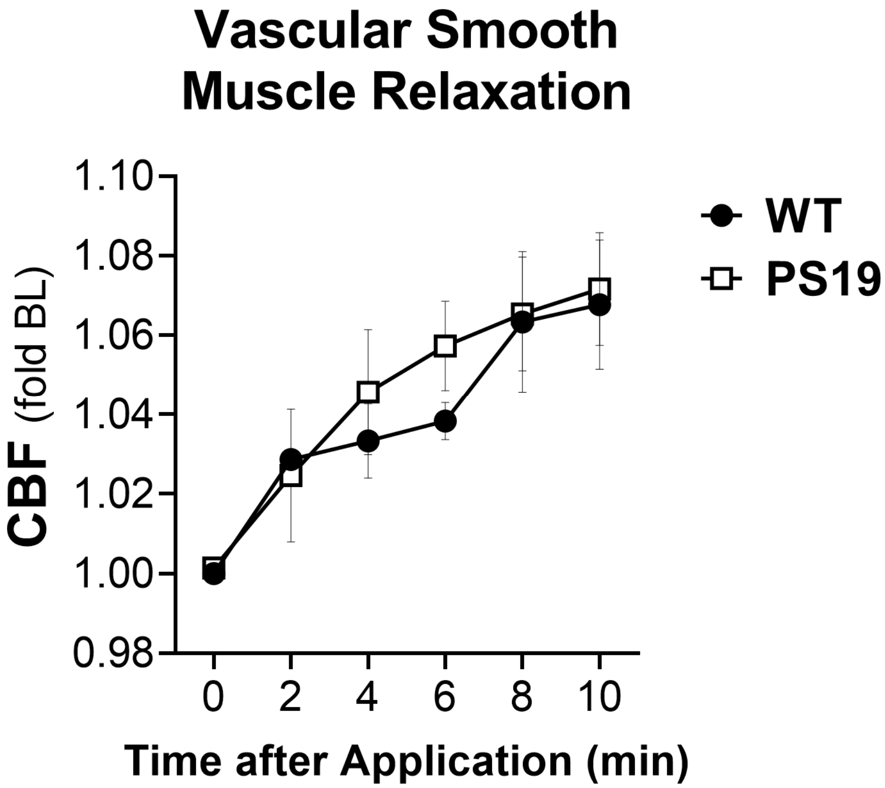

Supplementary Figure 4. Vascular smooth muscle function is unimpaired in male and female P301S(PS19) mice. Cerebral blood flow (CBF) responses in P301S(PS19) mice compared to wildtype (WT) littermates at 8-10 months of age of both sexes. Application of 20 μM sodium nitroprusside, a nitric oxide donor that acts on vascular smooth muscle to cause vasodilation, significantly increased cerebral blood flow in both WT and P301S(PS19) mice (F(1.863, 9.315)=14.72, RM ANOVA, P=0.0015). No significant differences in vascular smooth muscle function were found between WT and PS19 groups (F(1,4)=0.2724, RM ANOVA, P=0.6293. WT, n=3; PS19, n=3 mice). Data are means ± SEM.

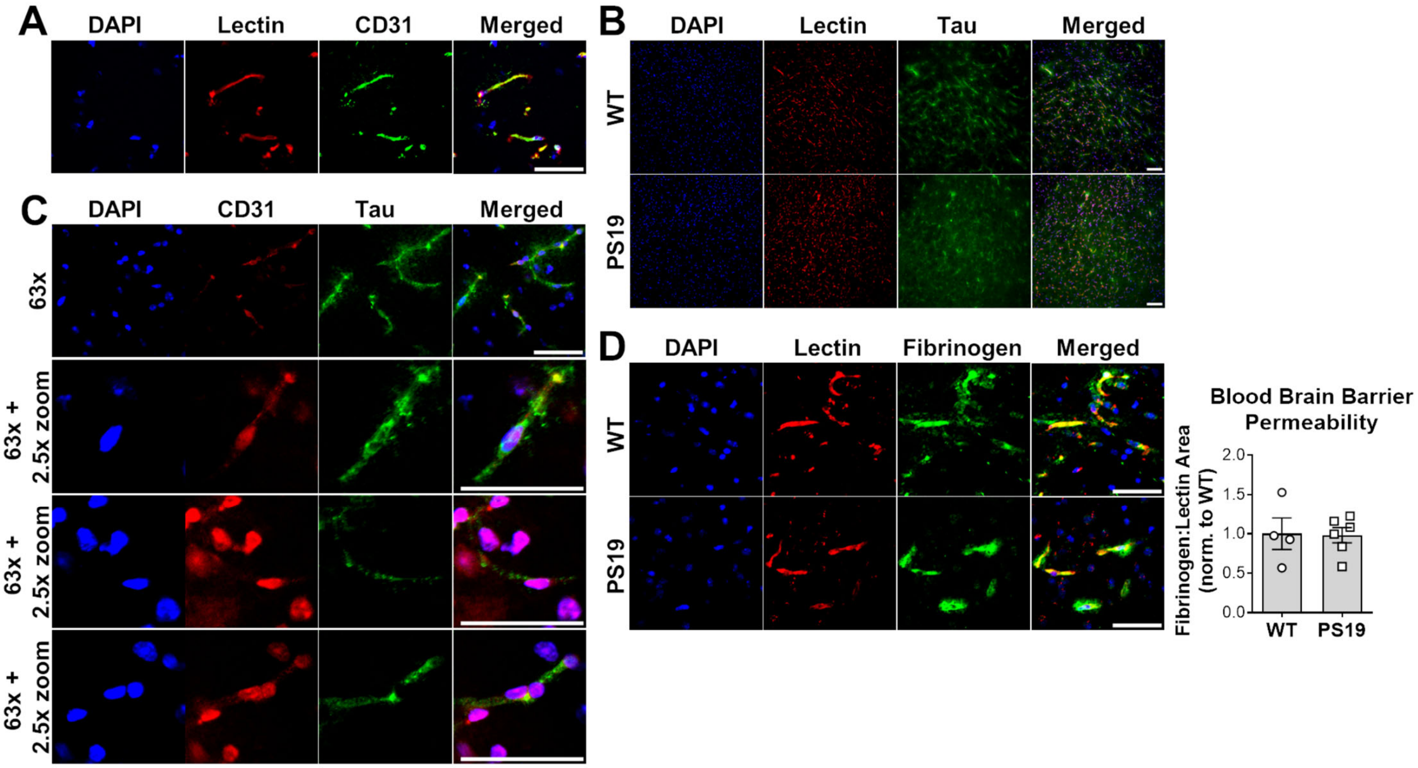

Supplementary Figure 5. Eight-month-old male and female P301S(PS19) mice display tau immunoreactivity in brain microvasculature without changes in blood brain barrier permeability. A, Representative images (63X) of P301S(PS19) cortical brain sections shows that lectin (red) stains endothelial cells of the brain microvasculature only as shown by its exclusive colocalization with CD31 (green) immunoreactivity with DAPI (blue). Scale bar is 50 µm. **B,** Representative low-magnification images (10X) of cortical brain sections showing total tau (Tau5, green) immunoreactivity in P301S(PS19) and WT brain microvasculature (lectin, red) at 8 months of age. **C,** Representative high-magnification images (63X and 63X+2.5X digital zoom) of cortical brain sections showing total tau total tau (Tau5, green) immunoreactivity in P301S(PS19) brain endothelial cells (CD31, red)) at 8 months of age. Scale bar is 50 µm. **D,** Representative images (63X) of brain microvasculature (lectin, red) and fibrinogen (green) cortical brain sections of P301S(PS19) and WT at 8 months of age. Scale bar is 50 µm. **E,** Quantification of ratio of fibrinogen area to lectin area as a measure of blood brain barrier permeability. No significant differences in blood brain barrier permeability were observed between WT and P301S(PS19) mice (unpaired, two-sided, Student's t test, $t(4.422)=0.09254$, $P=0.9303$. WT, n=4; PS19, n=6 mice). Data are means ± SEM.

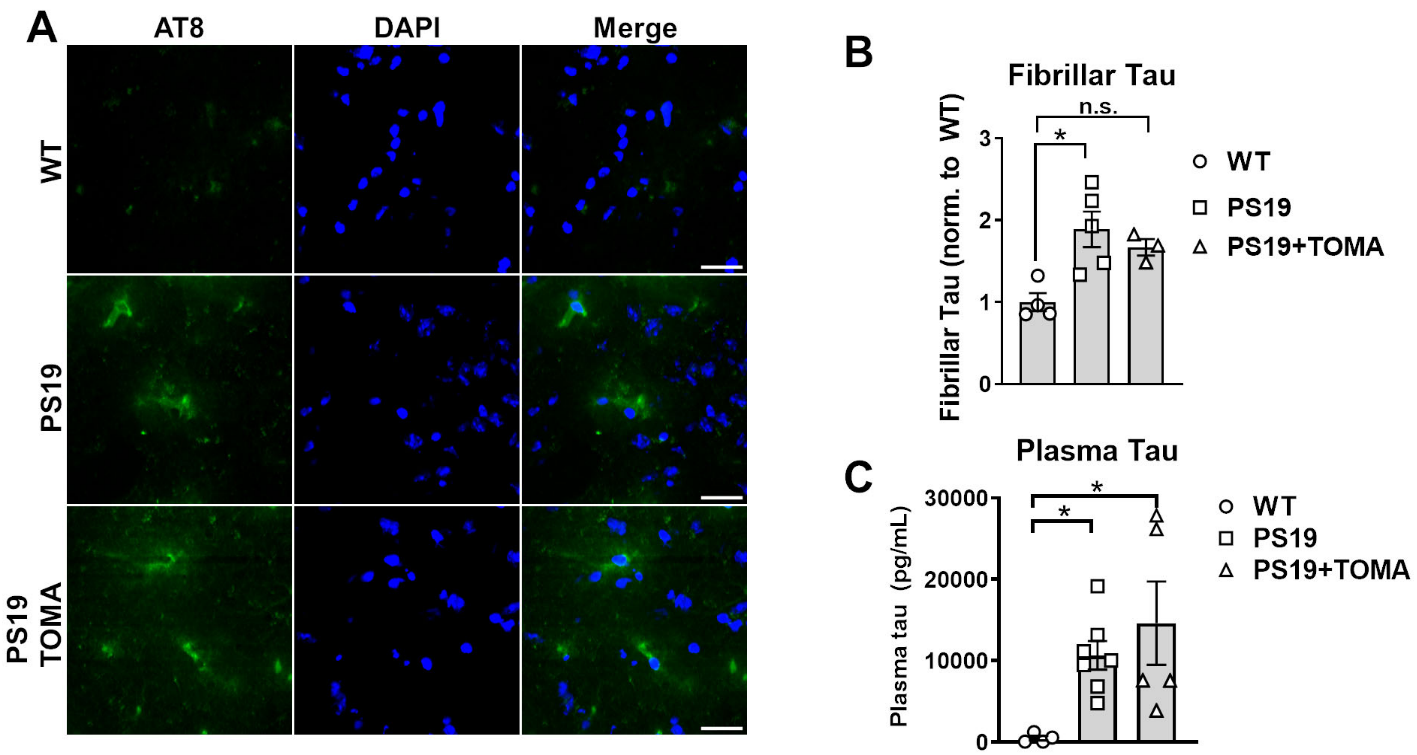

Supplementary Figure 6. Treatment with tau oligomer-specific monoclonal antibody (TOMA) does not reduce cortical brain fibrillar tau or total plasma tau in 12-month-old male and female P301S(PS19) mice. A, Representative images (63X) of cortical brain sections showing fibrillar tau immunoreactivity (phospho-tau, AT8, green) with DAPI (blue) in 12-month-old WT or P301S(PS19) male and female mice treated with tau oligomer-specific monoclonal antibody (TOMA) or same isotype IgG. Scale bar is 50 μm. **B,** Quantitative analysis of immunofluorescence data in **A** show a significant increase in fibrillar tau (AT8) in P301S(PS19) mice as compared to WT controls that is not reduced by TOMA ($F(2, 9) = 7.223$, ANOVA, *, $P<0.05$ by Tukey's. WT, n=4; PS19, n=5; PS19+TOMA, n=3 mice). **C,** Plasma levels of tau are significantly increased in P301S(PS19) mice as compared to WT controls at 12 months that is not significantly affected by TOMA ($H=8.474$, Kruskal-Wallis, $P=0.0067$; WT vs PS19, *, $P<0.05$; WT vs. PS19+TOMA, *, $P<0.05$ by Dunn's. n.s. indicates $p=0.07$ by Dunn's. WT, n=4; PS19, n=8; PS19+TOMA, n=5). Data are means ± SEM. For post-hoc analyses, a lack of a specific P value in the legend reflects the information reported by GraphPad Prism Version 9.4.0.
