# Supplementary material for: Soluble pathogenic tau enters brain vascular endothelial cells and drives cellular senescence and brain microvascular dysfunction in a mouse model of tauopathy
Source: Nat Commun. 2023 Apr 25;14:2367. doi: 10.1038/s41467-023-37840-y (PMC10126555; doi:10.1038/s41467-023-37840-y)
Supplement: Supplementary file 3 — Reporting Summary [file 41467_2023_37840_MOESM3_ESM.pdf]

nature portfolio

Corresponding author(s): Veronica Galvan

Last updated by author(s): Mar 10, 2023

Reporting Summary

Nature Portfolio wishes to improve the reproducibility of the work that we publish. This form provides structure for consistency and transparency in reporting. For further information on Nature Portfolio policies, see our Editorial Policies and the Editorial Policy Checklist.

Statistics

For all statistical analyses, confirm that the following items are present in the figure legend, table legend, main text, or Methods section.

n/a	Confirmed	
☐	☒	The exact sample size (*n*) for each experimental group/condition, given as a discrete number and unit of measurement
☐	☒	A statement on whether measurements were taken from distinct samples or whether the same sample was measured repeatedly
☐	☒	The statistical test(s) used AND whether they are one- or two-sided<br>*Only common tests should be described solely by name; describe more complex techniques in the Methods section.*
☐	☒	A description of all covariates tested
☐	☒	A description of any assumptions or corrections, such as tests of normality and adjustment for multiple comparisons
☐	☒	A full description of the statistical parameters including central tendency (e.g. means) or other basic estimates (e.g. regression coefficient) AND variation (e.g. standard deviation) or associated estimates of uncertainty (e.g. confidence intervals)
☐	☒	For null hypothesis testing, the test statistic (e.g. *F*, *t*, *r*) with confidence intervals, effect sizes, degrees of freedom and *P* value noted<br>*Give P values as exact values whenever suitable.*
☒	☐	For Bayesian analysis, information on the choice of priors and Markov chain Monte Carlo settings
☒	☐	For hierarchical and complex designs, identification of the appropriate level for tests and full reporting of outcomes
☒	☐	Estimates of effect sizes (e.g. Cohen's *d*, Pearson's *r*), indicating how they were calculated

Our web collection on statistics for biologists contains articles on many of the points above.

Software and code

Policy information about availability of computer code

Data collection	Zeiss LSM 780 microscope software ZEN 2012 SP2 (black) 11.0.1.190, NIS-Elements 5.11.01 (Nikon), Compass for SW software 6.0.0 (ProteinSimple), SDS 2.3 software (Applied Biosystems), ,Image Studio Lite 5.2 (Licor), Harmony High-Content Imaging Data Collection and Analysis Platform 4.8, Polar Star Omega 5.70 (BMG Labtech), Multimode Nanoscope IIIa, Zoe Fluorescent Cell Imager Software 002.257.011215, Lab Chart 8.0.7.
Data analysis	Image J Fiji (NIH), Compass for SW 6.0.0 (Protein Simple), Harmony High-Content Imaging Data analysis Platform 4.8 (Perkin Elmer), GraphPad Prism 9.4.0, SPIP software 6.0.13 (Image Metrology), SDS 2.3 software (Applied Biosystems)

For manuscripts utilizing custom algorithms or software that are central to the research but not yet described in published literature, software must be made available to editors and reviewers. We strongly encourage code deposition in a community repository (e.g. GitHub). See the Nature Portfolio guidelines for submitting code & software for further information.

Data

Policy information about availability of data

All manuscripts must include a data availability statement. This statement should provide the following information, where applicable:

- Accession codes, unique identifiers, or web links for publicly available datasets
- A description of any restrictions on data availability
- For clinical datasets or third party data, please ensure that the statement adheres to our policy

Source data is included with the submission.

Human research participants

Policy information about studies involving human research participants and Sex and Gender in Research.

Reporting on sex and gender	*Use the terms sex (biological attribute) and gender (shaped by social and cultural circumstances) carefully in order to avoid confusing both terms. Indicate if findings apply to only one sex or gender; describe whether sex and gender were considered in study design whether sex and/or gender was determined based on self-reporting or assigned and methods used. Provide in the source data disaggregated sex and gender data where this information has been collected, and consent has been obtained for sharing of individual-level data; provide overall numbers in this Reporting Summary. Please state if this information has not been collected. Report sex- and gender-based analyses where performed, justify reasons for lack of sex- and gender-based analysis.*
Population characteristics	*Describe the covariate-relevant population characteristics of the human research participants (e.g. age, genotypic information, past and current diagnosis and treatment categories). If you filled out the behavioural & social sciences study design questions and have nothing to add here, write "See above."*
Recruitment	*Describe how participants were recruited. Outline any potential self-selection bias or other biases that may be present and how these are likely to impact results.*
Ethics oversight	*Identify the organization(s) that approved the study protocol.*

Note that full information on the approval of the study protocol must also be provided in the manuscript.

Field-specific reporting

Please select the one below that is the best fit for your research. If you are not sure, read the appropriate sections before making your selection.

[x] Life sciences [ ] Behavioural & social sciences [ ] Ecological, evolutionary & environmental sciences

For a reference copy of the document with all sections, see nature.com/documents/nr-reporting-summary-flat.pdf

Life sciences study design

All studies must disclose on these points even when the disclosure is negative.

Sample size	For in vitro studies, post-hoc power analyses (G*Power) showed a probability of a Type 2 error was close to zero, thus all our in vitro studies were adequately powered to detect differences. Group size calculations (G*Power) for in vivo studies showed that n=4 resulted in 90% power to detect differences of 30% between experimental group means at $\alpha = 0.05$ using ANOVA and Tukey's post hoc tests, or Kruskal-Wallis and Dunn's. Sample sizes for all experiments are included in the figure legends.
Data exclusions	We pre-established our inclusion criteria such that all data points collected in all our experiments were not statistical outliers, i.e. were within the range of variance defined in Grubb's test. Thus, all data points collected in all our experiments were subjected to Grubb's test, setting alpha=0.05. Grubb's tests were ran using GraphPad Prism 9.4.0. Only data points identified as statistical outliers were excluded. We did not find more than 2 outlier data points in any of our experiments.
Replication	For all in vitro studies, we performed at least 3 independent experiments to demonstrate reproducibility. All data shown represent at least three independent experiments that yielded comparable results. No independent experiments were excluded. For in vivo studies with mice, we performed 3 independent experiments using separate cohorts of animals (4, 8, 12 mo.).
Randomization	Transgenic animals were randomized to treatment groups blindly by animal identifier and age. Animals in different treatment groups or genotype were randomized to test on different days, so that all groups were equally represented on each testing day.
Blinding	All operators were blinded to treatment and genotype when testing and only animal identifiers were used. For all capillary electrophoresis immunoassays, the Compass software (ProteinSimple) defines peaks and performs quantitative analyses of peak area in an unbiased, automated fashion, without involvement of the operator.

Reporting for specific materials, systems and methods

We require information from authors about some types of materials, experimental systems and methods used in many studies. Here, indicate whether each material, system or method listed is relevant to your study. If you are not sure if a list item applies to your research, read the appropriate section before selecting a response.

Materials & experimental systems

n/a	Involved in the study
☐	☒ Antibodies
☐	☒ Eukaryotic cell lines
☒	☐ Palaeontology and archaeology
☐	☒ Animals and other organisms
☒	☐ Clinical data
☒	☐ Dual use research of concern

Methods

n/a	Involved in the study
☒	☐ ChIP-seq
☒	☐ Flow cytometry
☒	☐ MRI-based neuroimaging

Antibodies

Antibodies used

Antibody information is also supplied in Supplementary Table 1.
Antibody Supplier Catalog # Lot Clone Species Application/Dilution
IF/ICC Dilution Traditional WB CE Immunblots
β-Actin Cell Signaling Technology 4970S 15 13E5 Rabbit 1:1000 1:200
CD31 BD Biosciences 553370 2146504 MEC 13.3 Mouse 1:500
eNOS R&D AF950 BBu0417051 Goat 1:500
phospho-eNOS (S1176/1177) BD Biosciences 612392 9044662 19/eNOS/S1177 mouse 1:50
phospho-eNOS (T495) BD Biosciences 612706 5329915 31/eNOS(pT495) Mouse 1:50
eNOS Millipore 07-520 2708538 Rabbit 1:75
Fibrinogen Dako Agilent A0080 15063 Rabbit 1:750
GAPDH Thermo Fisher Scientific PA1-987 UG28627 Rabbit 1:1000
Tau5 Millipore MAB361 3275077 Tau-5 Mouse 1:500 1:500 1:50
phospho-Tau (T231) Thermo Scientific, MN1040 PF202799 AT180 Mouse 1:50
phospho-Tau AT8 Thermo Fisher Scientific MN1020 VE2953712 AT8 Mouse 1:500
T22 (Oligomeric tau) Dr. Rakez Kayed Lab Rabbit 1:500
α-tubulin Cell Signaling Technology 2144S 6 Rabbit 1:1000
Acetyl-α-Tubulin Santa Cruz Biotechnology sc-23950 F1516 6-11B-1 Mouse 1:500 1:2000
β-tubulin Sigma Aldrich T4026 107M4801V TUB2.1 Mouse 1:1000
α/β tubulin Cell Signaling Technology 2148S 8 Rabbit 1:1000
V5 antibody Thermo Fisher Scientific R960-25 2024280 P/N 46-0705 Mouse 1:500
"Goat anti-Rabbit IgG (H+L) Cross-AdsorbedSecondary Antibody, Alexa Fluor™ 594" Invitrogen A11012 1933366 Goat 1:500
"Goat anti-Mouse IgG (H+L) Highly Cross-AdsorbedSecondary Antibody, Alexa Fluor™ 488" Invitrogen A11029 2120125 Goat 1:500
"Goat anti-Mouse IgG (H+L) Highly Cross-AdsorbedSecondary Antibody, Alexa Fluor™ 647" Invitrogen A21236 1229707 Goat 1:500
"Donkey anti-Goat IgG (H+L) Highly Cross-AdsorbedSecondary Antibody, Alexa Fluor™ Plus 488" Invitrogen A32814 UI289709 Donkey 1:500

Validation

Validation for antibodies was done by the supplier and validated by immunoassay in our laboratory. Below is the detailed antibody validation:
Antibody Supplier Catalog # Validation
β-actin Cell Signaling Technology 4970S >6,000 citations, runs at the correct molecular weight immunoassay
CD31 BD Biosciences 553370 The supplier routinely tests this antibody by flow cytometry
eNOS R&D AF950 Cited >8 times and it runs at the correct molecular weight immunoassay
phospho-eNOS S1176/1177 BD Biosciences 612392 The supplier tests this antibody in western blot routinely. And tested this antibody with Flow cytometry during the development phase
phospho-eNOS T495 BD Biosciences 612706 The antibody runs at the correct molecular weight immunoassay
eNOS Millipore 07-520 "This antibody detects level of eNOS/NOS III & has been published &validated for use in Western blot by the supplier"
Fibrinogen Dako Agilent A008002-2 We have validated this antibody previoulsy PMC5966773
GAPDH Thermo Fisher Scientific PA1-987 26 publised figuers and 47 references the antibody runs at the correct molecular weight immunoassay
Tau5 Millipore MAB361 The supplier validated this antibody for use in immunohistochemistry and Westren blot
phospho-tau T231 Thermo Scientific MN1040 > 274 References. the supplier validated that this antibody recognizes PHF-Tau and tangles, and Does not cross react with recombinant unphosphorylated human Tau
phospho-tau AT8 Thermo Fisher Scientific MN1020 >739 References, The supplier has verified this antibody by advanced verification methods using cell treatments to ensure that the antibody binds to the antigen stated
T22 (Oligomeric tau) Dr. Rakez Kayed Lab Designed and validated by Dr.Rakez Kayed lab, PMC4046102

α-tubulin Cell Signaling Technology 2144S >920 citations - runs at the correct molecular weight immunoassay<br>Acetyl-α-tubulin Santa Cruz Biotechnology sc-23950 > 297 citations, runs at the correct molecular weight immunoassay<br>β-tubulin Sigma Aldrich T4026 The supplier tested this antibody with Standard antibody validation processes and Enhanced Validation process using independent Antibodies.<br>α/β tubulin Cell Signaling Technology 2148S >640 citation, runs at the correct molecular weight immunoassay<br>V5 antibody Thermo Fisher Scientific R960-25 The supplier validated this antibody using Western blot (against 25 ng of recombit Positope™ protein).<br>Goat anti-Rabbit IgG (H+L) Cross-Adsorbed Secondary Antibody, Alexa Fluor™ 594 Invitrogen A11012 40 Published Figures, 2326 References, secondary only was nonreactive<br>Goat anti-Mouse IgG (H+L) Highly Cross-Adsorbed Secondary Antibody, Alexa Fluor™ 488 Invitrogen A11029 39 Published Figures, 4630 References, secondary only was nonreactive<br>Goat anti-Mouse IgG (H+L) Highly Cross-Adsorbed Secondary Antibody, Alexa Fluor™ 647 Invitrogen A21236 9 Published Figures, 979 References, secondary only was nonreactive<br>Donkey anti-Goat IgG (H+L) Highly Cross-Adsorbed Secondary Antibody, Alexa Fluor™ Plus 488 Invitrogen A32814 142 References, secondary only was nonreactive

Eukaryotic cell lines

Policy information about cell lines and Sex and Gender in Research

Cell line source(s)	All in vitro experiments were performed on primary human brain microvascular endothellial cells (HBEC). No cell lines were used in our studies. Primary human brain microvascular endothelial cells were purchased from Cell Biologics, Cat# H-6023.
Authentication	From Cell Biologics: Human Primary Brain Microvascular Endothelial Cells from Cell Biologics display typical cobblestone with large dark nuclei appearance under light microscopy. Cells are tested for expression of endothelial cell marker using antibody, CD31 (Catalog No. 550389, BD; CD31/PECAM-1 PE-conjugated Antibody, Catalog No. FAB3567P, R&D) or VE-Cadherin (FITC-VE-cadherin Catalog No. 560411, BD) by immunofluorescence staining or FACS. We also confirmed experimentally that the HBEC used in our experiments express endothelial nitric oxide synthase (eNOS) by immunoassay, another marker of endothelial cells
Mycoplasma contamination	From Cell Biologics: All cells test negative for mycoplasma, bacteria, yeast, and fungi. HIV-1, hepatitis B and hepatitis C are not detected for all donors and/or cell lots. Furthermore, HBEC were only cultured for </= 5 days in continuous culture and mycoplasma cannot grow in that short of time.
Commonly misidentified lines<br>(See ICLAC register)	No cell lines were used in our studies.

Animals and other research organisms

Policy information about studies involving animals; ARRIVE guidelines recommended for reporting animal research, and Sex and Gender in Research

Laboratory animals	Mice from in-house P301S(P19) colonies established and maintained using breeders obtained from JAX (B6;C3-Tg(Prnp-MAPT*P301S)PS19Vle/J), stock #008169) were used in the studies reported.
Wild animals	None
Reporting on sex	All studies reported were performed on groups that were a mixture of male and female mice, except for the 4 month-old groups (WT and PS19, 6 animals/group, all male) in Figure 4A. We were not powered to detect sex differences, however no trends were gleaned from the data collected from all other experimental groups, which are reported in Figures 4-6 and Supplementary Figures 4-6.
Field-collected samples	None
Ethics oversight	Experiments were performed with approval from the University of Texas Health San Antonio Institutional Animal Care and Use Committee (Animal Welfare Assurance Number A3345-01), which complies with the Animal Welfare Act, the Guide for the Care and Use of Laboratory Animals, the Public Health Service Policy on Humane Care and Use of Laboratory Animals, the US Government Principles for the Utilization and Care of Vertebrate Animals Used in Testing, Research, and Training, and the ARRIVE (Animal Research: Reporting In Vivo Experiments) guidelines for reporting animal experiments.

Note that full information on the approval of the study protocol must also be provided in the manuscript.
